# Fabrication of multifunctional metal–organic frameworks nanoparticles via layer-by-layer self-assembly to efficiently discover PSD95-nNOS uncouplers for stroke treatment

**DOI:** 10.1186/s12951-022-01583-7

**Published:** 2022-08-13

**Authors:** Yingying Ding, Yang Jin, Tao Peng, Yankun Gao, Yang Zang, Hongliang He, Fei Li, Yu Zhang, Hongjuan Zhang, Lina Chen

**Affiliations:** 1 School of Pharmacy, Nanjing Medical University, Nanjing, Jiangsu 211166 People’s Republic of China; 2 College of Economics and Management, Anhui Agricultural University, Hefei, Anhui 230036 People’s Republic of China; 3 Department of Pharmacy, Sir Run Run Hospital, Nanjing Medical University, Nanjing, Jiangsu 211166 People’s Republic of China

**Keywords:** multifunctional nanoparticles, Coordinative immobilization, Fluorescent sensitivity, PSD95-nNOS uncouplers

## Abstract

**Background:**

Disruption of the postsynaptic density protein-95 (PSD95)—neuronal nitric oxide synthase (nNOS) coupling is an effective way to treat ischemic stroke, however, it still faces some challenges, especially lack of satisfactory PSD95-nNOS uncouplers and the efficient high throughput screening model to discover them.

**Results:**

Herein, the multifunctional metal–organic framework (MMOF) nanoparticles as a new screening system were innovatively fabricated via layer-by-layer self-assembly in which His-tagged nNOS was selectively immobilized on the surface of magnetic MOF, and then PSD95 with green fluorescent protein (GFP-PSD95) was specifically bound on it. It was found that MMOF nanoparticles not only exhibited the superior performances including the high loading efficiency, reusability, and anti-interference ability, but also possessed the good fluorescent sensitivity to detect the coupled GFP-PSD95. After MMOF nanoparticles interacted with the uncouplers, they would be rapidly separated from uncoupled GFP-PSD95 by magnet, and the fluorescent intensities could be determined to assay the uncoupling efficiency at high throughput level.

**Conclusions:**

In conclusion, MMOF nanoparticles were successfully fabricated and applied to screen the natural actives as potential PSD95-nNOS uncouplers. Taken together, our newly developed method provided a new material as a platform for efficiently discovering PSD95-nNOS uncouplers for stoke treatment.

**Graphical Abstract:**

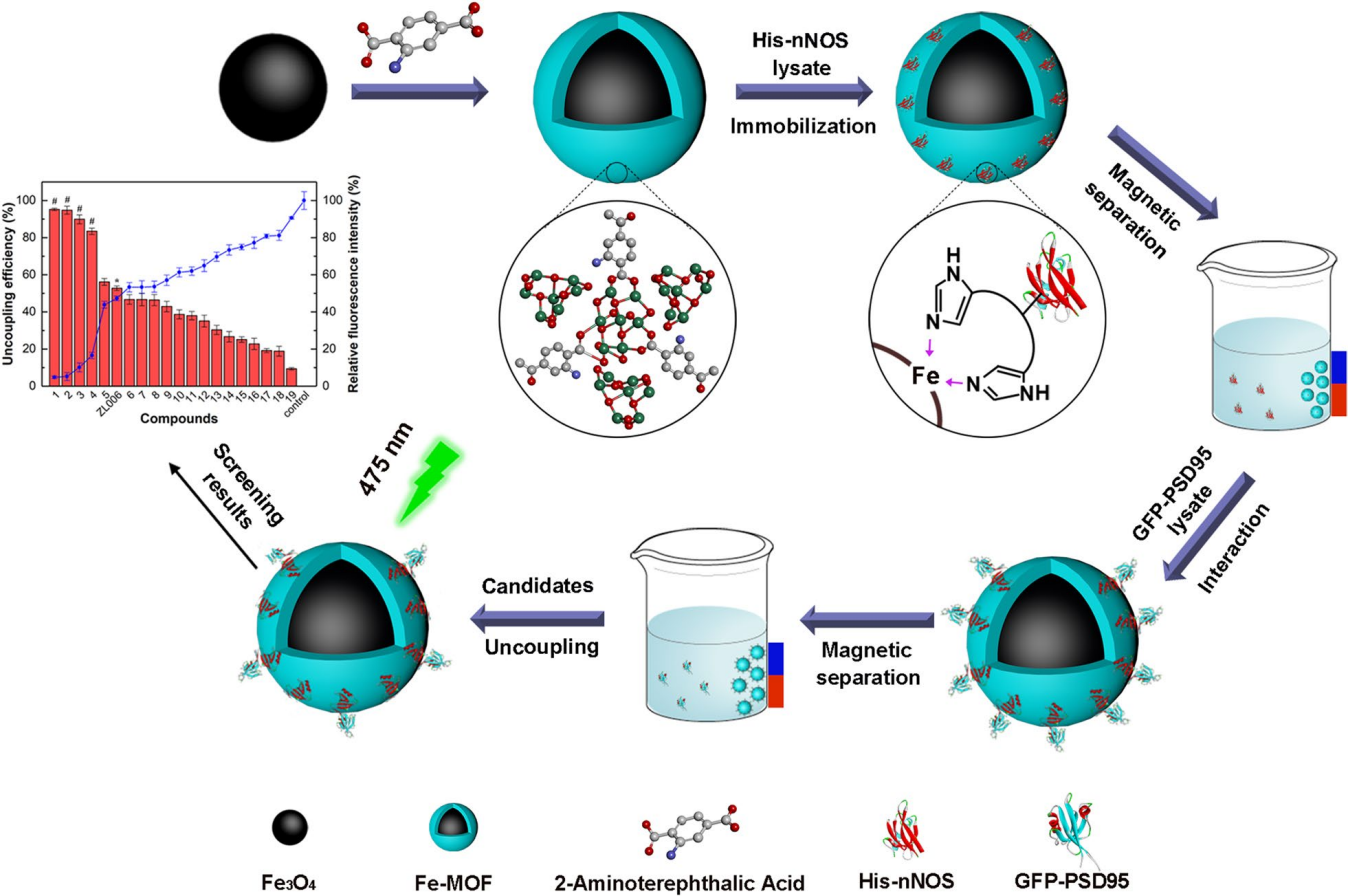

**Supplementary Information:**

The online version contains supplementary material available at 10.1186/s12951-022-01583-7.

## Introduction

Ischemic stroke, caused by the disruption of blood supply to the brain [1], is a disease with high morbidity, high recurrence rate, severe disability and alarming mortality accompanied by multiple complications [2, 3], which currently has become one of the main diseases that endanger public health [4, 5]. In the process of cerebral ischemia, the over-released glutamate excessively activates *N*-methyl-d-aspartate receptors (NMDARs) and thereby induces neuronal death [1, 6], which is caused by NMDAR-dependent neuronal nitric oxide synthase (nNOS) translocation from cytosol to membrane via the interaction of nNOS and postsynaptic density protein-95 (PSD95) [7]. In order to prevent cerebral ischemic injury and avoid the undesirable effects of directly blocking NMDAR and inhibiting nNOS, it is necessary to block nNOS translocation by dissociating the ischemia-induced PSD95–nNOS interaction [8–10]. Based on the mechanism above, ZL006 and IC87201 were designed and developed as PSD95-nNOS uncouplers [10–12]. However, fast metabolism and low permeability across the blood brain barrier limited their further clinical application [13], so novel PSD95-nNOS uncouplers need be exploited urgently.

It is a top priority to establish an efficient high throughput screening model to find out better PSD95-nNOS uncouplers. We had designed the molecularly imprinted polymers instead of nNOS and PSD95 as receptors to capture several uncouplers from complex samples like the extracts of natural medicines and Sanhuang Xiexin Decoction, whose potential dissociation of PSD95 and nNOS to treat stroke was demonstrated by the experiments on glutamate-injured PC12 cells and MCAO/R (middle cerebral artery occlusion and reperfusion) rats in vivo [14–16]. However, some problems such as high costs, complicated operations, poor reusability and stability of proteins still exist. Therefore, it is highly desirable to develop a convenient, highly sensitive and stable screening method in the discovery of uncouplers.

At present, metal–organic frameworks (MOFs), the well-known coordination polymers with three-dimensional pore structure, remarkable surface areas and porosity, adjustable pore size and diverse topological structures [17–21], have been a burgeoning research area of employing MOFs for the immobilization of proteins, which shows promise for meeting real-world challenges [22–26]. Thus, it is very attractive to immobilize PSD95-nNOS on MOFs to construct the in vitro screening system. For MOFs, various kinds of immobilizing proteins on/into MOFs have been reported, such as adsorption, covalence, entrapment, cross-linking and affinity. The strategy of affinity is to connect a functional group on a support with the affinity tag on a protein sequence, which can achieve oriented and site-specific immobilization of proteins [27]. For example, His tag could specifically bind to the coordination unsaturated metal sites on MOFs (Ni^2+^, Cu^2+^, Fe^3+^, etc.) to separate the target protein with His tag from the general proteins without His tag [28]. According to the principle, bovine hemoglobin rich in His tag is successfully isolated from bovine blood samples [29], and the His-tagged-enzyme-MOFs biocomposites are prepared for ATP regeneration from AMP [30]. In view of the biological feasibility of this method, His tagged nNOS would selectively bind nNOS to MOFs and then specifically couple with PSD95 to establish a model for screening PSD95-nNOS uncouplers in vitro, meanwhile, the combination of PSD95-nNOS-MOF also provides an approach of purifying nNOS and PSD95 from the crude protein solution. In addition, magnetic separation can simplify the operation and speed up the separation of samples for repeated use [31]. Therefore, magnetic-MOF is first designed to bind His-tagged nNOS and then with PSD95 to construct the multifunctional nanoparticles.

When PSD95-nNOS are immobilized on MOFs, it is significant to detect the proteins content, especially the uncoupled PSD95. Currently, western blot, mass spectrometry and enzyme-linked immunosorbent assay (ELISA) are common techniques for protein detection [32]. Considering their complicated operations and the expensive cost [15, 33, 34], another rapid and sensitive method need be constructed. In recent years, fluorescent tags such as green fluorescent protein (GFP), red fluorescent protein (RFP) and yellow fluorescent protein (YFP) are commonly used in protein optical detection to enhance the detection sensitivity [35, 36], furthermore, fluorescence-based technologies have shown great characteristics of high sensitivity, rapid response and easy operation for high-throughput screening [37], therefore, it is a good choice to apply fluorescent tags to label PSD95 for more efficient detection.

**Scheme 1:**
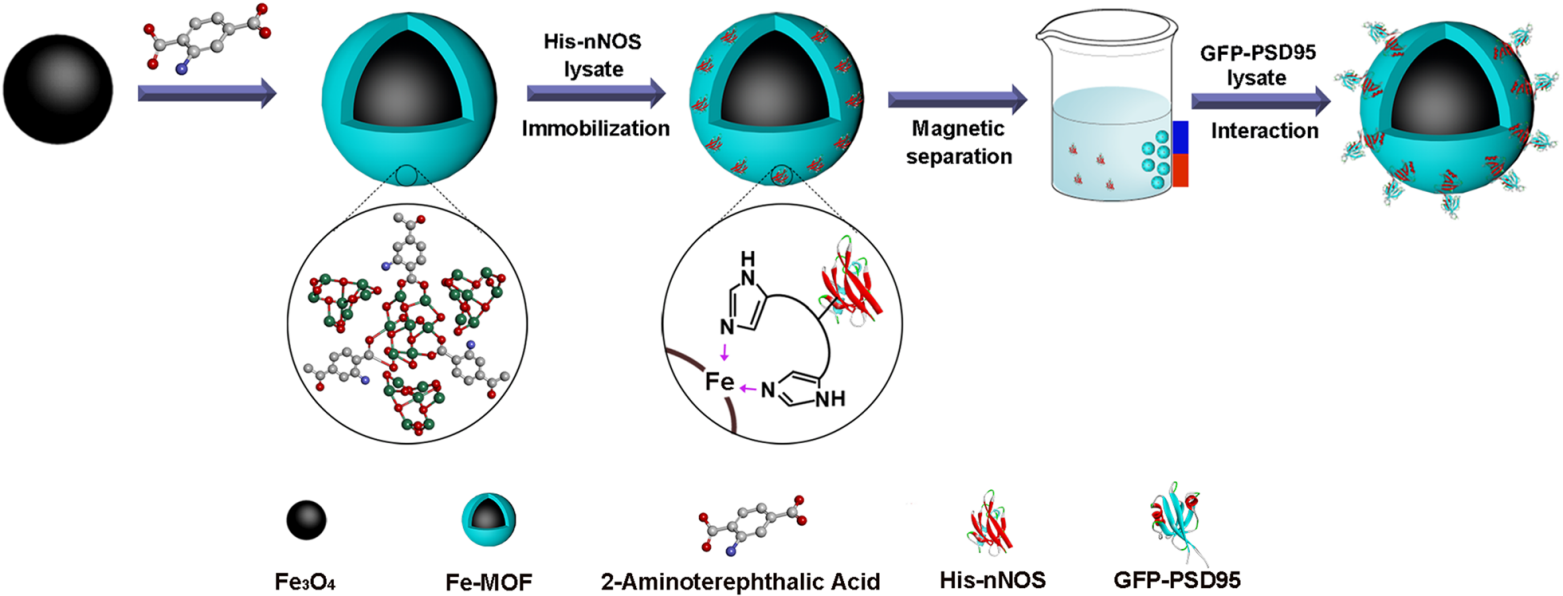
Schematic illustration of the synthesis procedure for PSD95-nNOS/Fe-MOF

As shown in Scheme 1, we first established a method for rapidly and sensitively screening the potential PSD95-nNOS uncouplers based on magnetic PSD95-nNOS/Fe-MOF as MMOF nanoparticles, which were layer-by-layer self-assembled through selectively immobilizing His-nNOS and GFP-PSD95 on the surface of magnetic Fe-MOF. The biocomposites were successfully prepared and systematically characterized, and its reusability, stability and anti-interference ability were all evaluated. The MMOF nanoparticles could be easily separated from uncoupling PSD95 by magnet after interacting with the uncouplers, and the fluorescent intensities were determined to assay the uncoupling efficiency at high throughput level. To be excited, the results demonstrated that this screening model could pick out the natural actives as the potential PSD95-nNOS uncouplers. In conclusion, PSD95-nNOS/Fe-MOF nanoparticles as a new material have been successfully developed and this new methodology will contribute to the discovery of PSD95-nNOS uncouplers by screening the candidates at high throughput level.

## Results and discussion

### Optimum conditions for His-nNOS immobilization

In order to investigate which MOF had the highest loading efficiency, we synthesized the other MOFs as shown in Table 1. We chose Fe^3+^, Cu^2+^ and Zr^4+^ as the metal center because of their high His-tag affinity and potential for biomedical applications [28]. Meanwhile, SBA-15 was inspected together since it’s a material with good adsorption performance proved by our previous report [38]. The detailed information about the synthesis of the materials and the procedure of immobilizing nNOS by different materials was shown in the “Additional file 1”. It can be concluded that magnetic Fe-MOF had the highest loading efficiency (about 92%), which showed superior adsorption performance compared with other materials (Table 1). In addition, SBA-15 mesoporous material exhibited high loading efficiency due to its large pore volume to hold more proteins. However, His-nNOS was so weakly combined with SBA-15 by physical adsorption that it could be easily eluted, furthermore, magnetic Fe-MOF could be separated by magnetism and was easy to operate and reuse. Therefore, magnetic Fe-MOF was chosen as the best immobilization material.

**Table 1:** Comparison of different immobilization materials on loading efficiency

Materials	Main reactant	Main reactant	Loading efficiency (%)
Magnetic Fe-MOF	Fe_3_O_4_	2-Aminoterephthalic acid	92.26 ± 0.55
Fe-COOH-MOF	Fe_3_O_4_-COOH	2-Aminoterephthalic acid	48.06 ± 1.83
UiO-66-NH_2_	ZrCl_4_	2-Aminoterephthalic acid	41.90 ± 1.03
Cu-BDC	Cu(NO_3_)_2_·2.5H_2_O	2-Aminoterephthalic acid	33.72 ± 1.02
HKUST-1	Cu(NO_3_)_2_·2.5H_2_O	Trimesic acid	63.70 ± 3.38
Zr-fum	ZrCl_4_	Fumaric acid	50.66 ± 1.32
SBA-15	Pluronic P123	Tetraethyl orthosilicate	80.67 ± 0.68

In order to acquire higher loading efficiency of multifunctional nanoparticles, the immobilization time, mass ratio of magnetic Fe-MOF to nNOS were optimized. The detailed operation for optimization was shown in the “Additional file 1”.

**Fig. 1:**
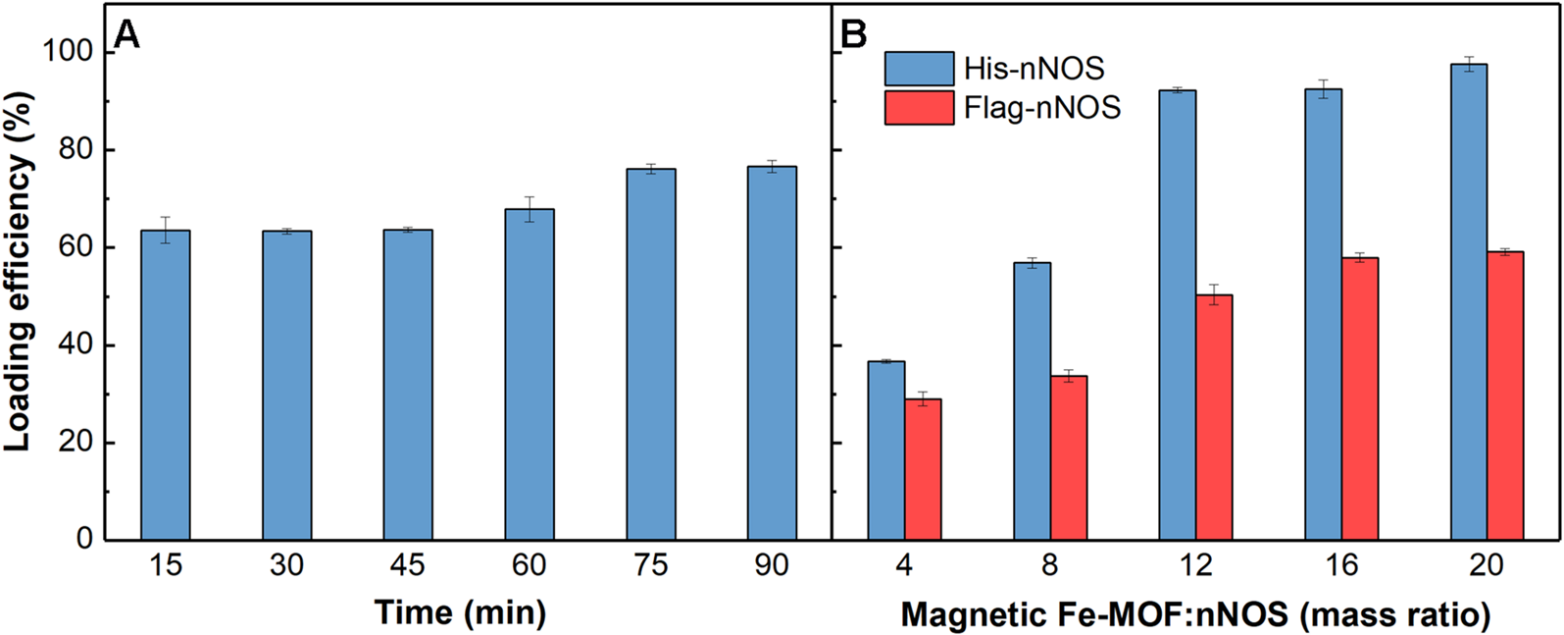
Effects of immobilization time (**A**) and mass ratio of magnetic Fe-MOF to nNOS (**B**) on loading efficiency

The effect of immobilization time on the loading efficiency was explored. The loading efficiency gradually increased with the extension of immobilization time, as described in Fig. 1A. When magnetic Fe-MOF and His-nNOS were incubated for 75 min, the loading efficiency tended to be stable. Therefore, 75 min was selected as the best immobilization time.

The influence of the mass ratio of magnetic Fe-MOF to nNOS with different tags on loading efficiency was investigated in Fig. 1B. No matter what the mass ratio of magnetic Fe-MOF to nNOS was, the loading efficiency of His-nNOS was higher than that of Flag-nNOS, which again proved the specific binding of His-nNOS to magnetic Fe-MOF. Meanwhile, when the mass ratio of magnetic Fe-MOF to His-nNOS was 12:1, the higher loading efficiency could be maintained without the waste of magnetic Fe-MOF. Therefore, we chose 12:1 as the optimal mass ratio of magnetic Fe-MOF to His-nNOS.

### Characterization

**Fig. 2:**
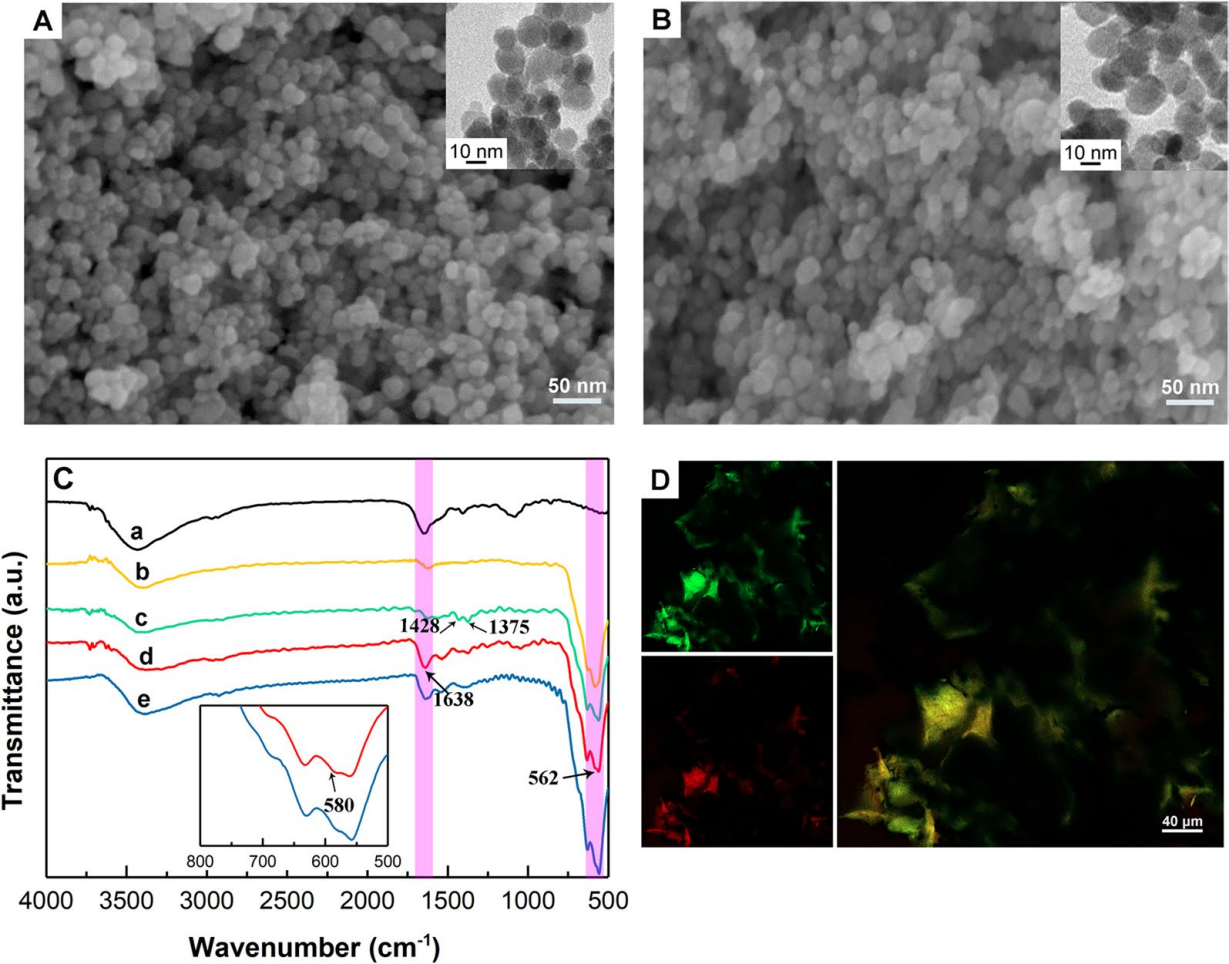
SEM images of (**A**) magnetic Fe-MOF (the inset is the TEM image of magnetic Fe-MOF) and (**B**) His-nNOS/Fe-MOF (the inset is the TEM image of His-nNOS/Fe-MOF), (**C**) FT-IR spectra of (**a**) Free His-nNOS, (**b**) Fe_3_O_4_, (**c**) magnetic Fe-MOF, (**d**) His-nNOS/Fe-MOF and (**e**) Flag-nNOS/Fe-MOF (the inset is the details of curve (**d**) and (**e**) from 500 to 800 cm^−1^), (**D**) the images of PSD95-nNOS/Fe-MOF by confocal laser scanning microscopy

The morphological structure of magnetic Fe-MOF and His-nNOS/Fe-MOF were characterized by SEM and TEM. Magnetic Fe-MOF was spherical and monodisperse with a particle size of 10–20 nm, as shown in Fig. 2A, B. In comparison to the other materials, the particle size of PSD95-nNOS/Fe-MOF was the smallest (Additional file 1: Table S1), which may provide a larger specific surface area and allow more proteins to be immobilized. This may also be the reason why the loading efficiency of this MOF was better than that of other MOFs.

The FT-IR spectra of free His-nNOS, Fe_3_O_4_, magnetic Fe-MOF, His-nNOS/Fe-MOF, Flag-nNOS/Fe-MOF were displayed in Fig. 2C. The typical bond at 562 cm^−1^ corresponded to the vibration of Fe–O in Fe_3_O_4_. According to the comparison with Fe_3_O_4_, the peaks at 1428 and 1375 cm^−1^ attributed to the symmetric stretching vibration of carboxylate group of NH_2_-BDC, which indicated the successful synthesis of magnetic Fe-MOF [30], as shown in Fig. 2C (c). The absorption peak at 1638 cm^−1^ attributed to the C=O in the amide, which was consistent with the spectra of His-nNOS in Fig. 2C (d) and (e). Meanwhile, the peak at 580 cm^−1^ of His-nNOS/Fe-MOF was likely to be associated with the Fe–N bond, while the peak at 580 cm^−1^ of Flag-nNOS/Fe-MOF was unapparent, demonstrating the coordinative binding of imidazole function in His-tags to the unsaturated metal sites on the external surface of magnetic Fe-MOF.

The His-nNOS was labelled by Rhodamine B (RhB) and then used to prepare PSD95-RhB-nNOS/Fe-MOF. The detailed information of the synthesis of PSD95-RhB-nNOS/Fe-MOF was presented in the “Additional file 1”. The prepared PSD95-RhB-nNOS/Fe-MOF was observed under a confocal laser scanning microscopy. The red and green fluorescence could be observed, which independently represented the RhB-labelled His-nNOS and GFP-PSD95, as demonstrated in Fig. 2D. The results well verified that both His-nNOS and GFP-PSD95 had been successfully immobilized on magnetic Fe-MOF.

**Fig. 3:**
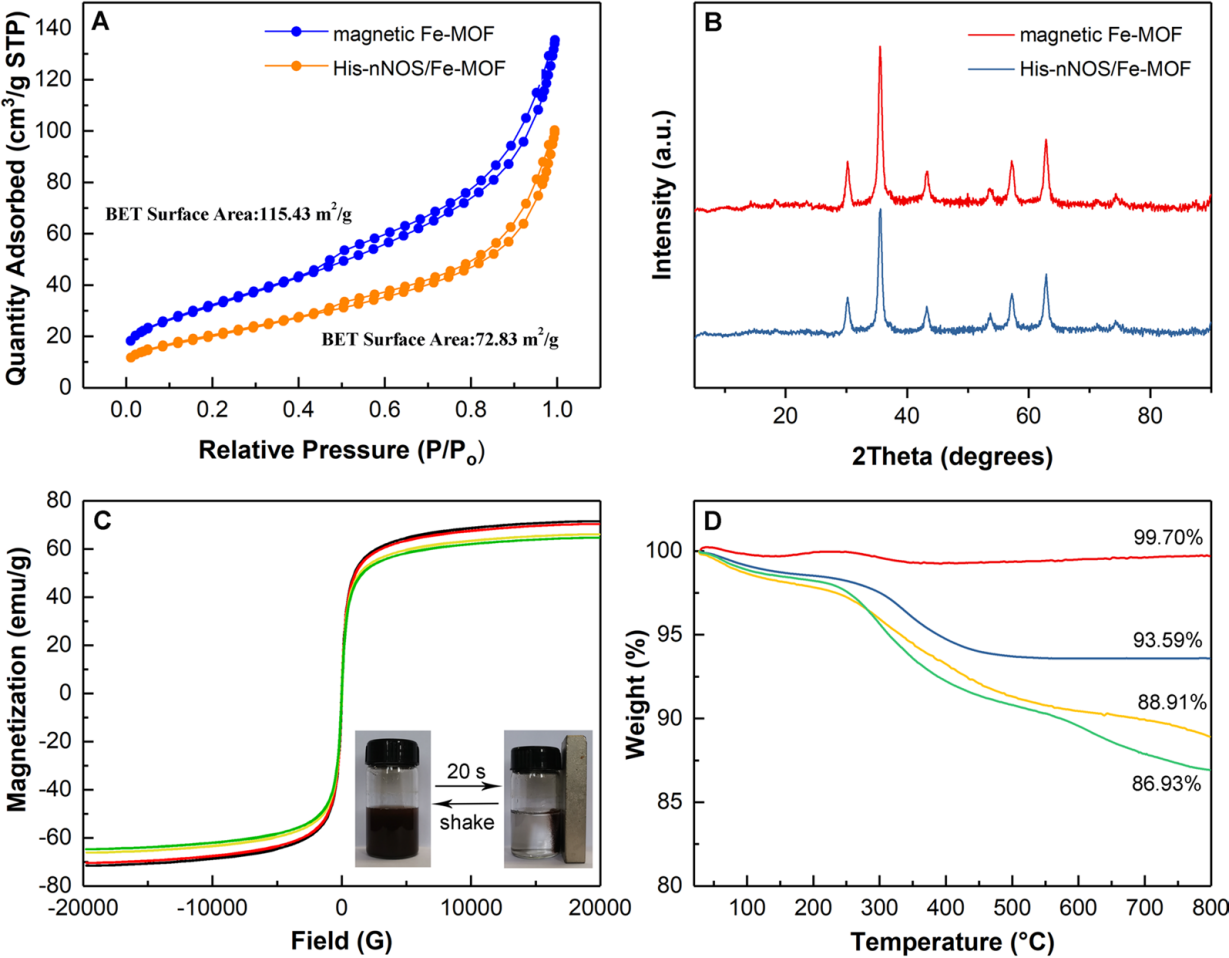
**A** N_2_ sorption isotherms of magnetic Fe-MOF and His-nNOS/Fe-MOF, (**B**) XRD patterns of magnetic Fe-MOF and His-nNOS/Fe-MOF, (**C**) magnetic analysis of (black) Fe_3_O_4_, (red) magnetic Fe-MOF, (yellow) His-nNOS/Fe-MOF and (green) PSD95-nNOS/Fe-MOF (the inset showed the rapid separation of PSD95-nNOS/Fe-MOF), and (**D**) thermogravimetric weight loss curve of (red) Fe_3_O_4_, (blue) magnetic Fe-MOF and (yellow) His-nNOS/Fe-MOF and (green) PSD95-nNOS/Fe-MOF

To further demonstrate the immobilization of His-nNOS, N_2_ sorption isotherms of magnetic Fe-MOF and His-nNOS/Fe-MOF were analyzed in Fig. 3A. His-nNOS/Fe-MOF had a BET surface area of 72.83 m^2^/g, which was smaller than that of magnetic Fe-MOF (115.43 m^2^/g). The decrease may be attributed to the attachment of His-nNOS on the outer surface of magnetic Fe-MOF, thus leading to the occupation and blocking of the pores of magnetic Fe-MOF [39]. Besides, compared to pure Fe_3_O_4_ (with a BET surface of 19.2 m^2^/g) [29], magnetic Fe-MOF enabled more active sites to be exposed and obtained larger BET surface to immobilize His-nNOS.

XRD patterns of magnetic Fe-MOF and His-nNOS/Fe-MOF were shown in Fig. 3B. Magnetic Fe-MOF and His-nNOS/Fe-MOF had the identical diffraction peaks, which indicated that the immobilization of His-nNOS couldn’t influence the crystal structure of magnetic Fe-MOF and confirmed the stability of magnetic Fe-MOF.

The magnetic properties were evaluated, as shown in Fig. 3C. The saturation magnetizations of Fe_3_O_4_, magnetic Fe-MOF, His-nNOS/Fe-MOF and PSD95-nNOS/Fe-MOF were detected as follows: 71.51, 70.40, 66.09, 64.70 emu/g, and the gradual decrease in magnetism could be put down to the combination of the organic linker 2-aminoterephthalic acid, as well as the immobilization of His-nNOS and the interaction of GFP-PSD95. Furthermore, although the saturation magnetization of PSD95-nNOS/Fe-MOF was the lowest, it was still strong enough to be rapidly separated by magnet in 20 s according to the inset.

Thermal gravimetric curves showed the weight loss of Fe_3_O_4_, magnetic Fe-MOF, His-nNOS/Fe-MOF and PSD95-nNOS/Fe-MOF was 0.3%, 6.41%, 11.09%, 13.07%, respectively (Fig. 3D). The slight loss of Fe_3_O_4_ could be attributed to the elimination of residual solvents while the increased weight loss of magnetic Fe-MOF resulted from the decomposition of the organic ligands. From the curve of His-nNOS/Fe-MOF and PSD95-nNOS/Fe-MOF, the further weight loss may be assigned to the disintegration of His-nNOS and GFP-PSD95. The gradual increase in weight loss confirmed the layer cracking of the attached materials. The above results further demonstrated that PSD95-nNOS/Fe-MOF particles were successfully layer-by-layer assembled.

**Fig. 4:**
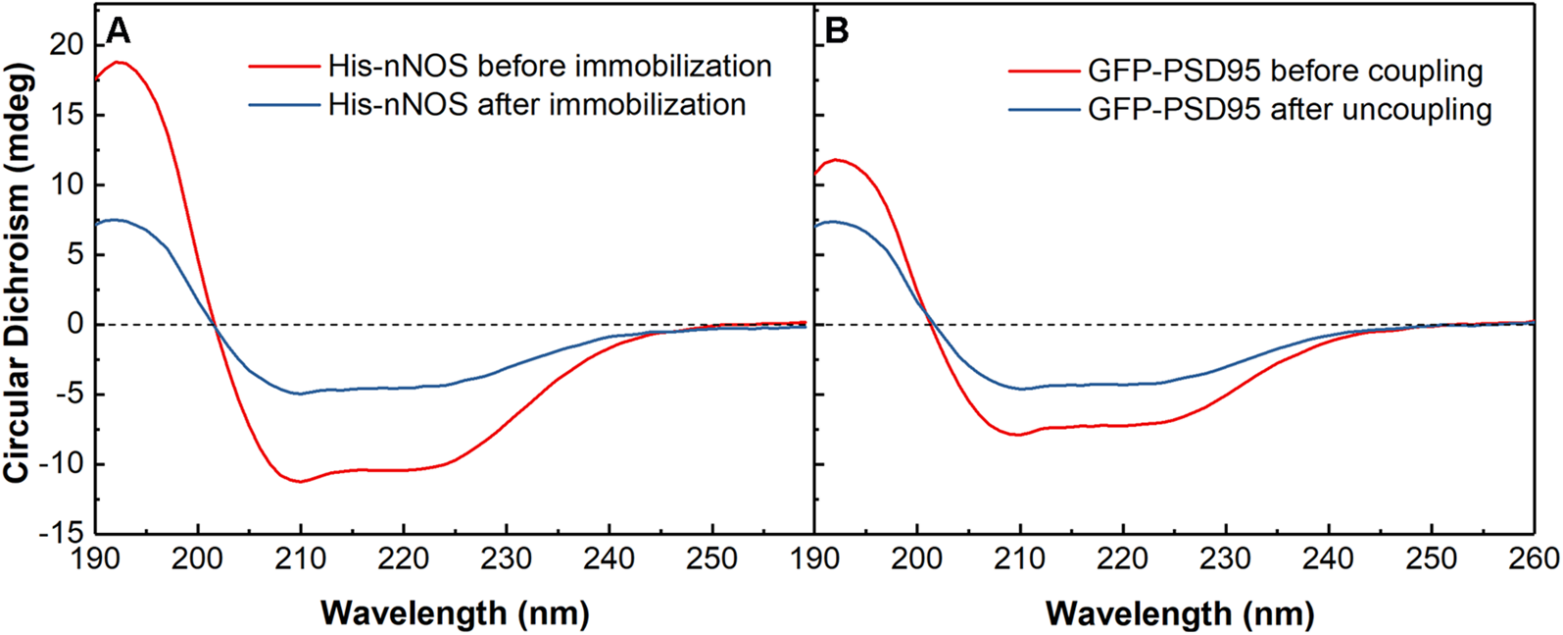
Circular dichroism spectra of His-nNOS and GFP-PSD95

The circular dichroism of His-nNOS and GFP-PSD95 was analyzed to investigate the stability of the proteins. According to the requirement and operability of the detected samples, all of them were prepared in solution. The His-nNOS before immobilization and GFP-PSD95 before coupling separately meant the initial protein solutions without any operations. When His-nNOS/Fe-MOF was eluted by imidazole buffer (100 mM), the collected His-nNOS solution was obtained as His-nNOS after immobilization. While PSD95-nNOS/Fe-MOF interacted with ZL006, the dissociated GFP-PSD95 was acquired as GFP-PSD95 after uncoupling. The circular dichroism bands were assigned to the β-sheet (195 nm) and α-helix (208 nm and 222 nm) of the proteins respectively, [40] and the circular dichroism spectra revealed that the secondary structures of two proteins remained unchanged, as described in Fig. 4. In addition, the decrease of circular dichroism signal intensity for the treated proteins was related to the decline of their concentrations [41].

The western blot analysis of His-nNOS and GFP-PSD95 was shown in Additional file 1: Fig. S1. It could be seen that the recombinant plasmids were constructed and the proteins His-nNOS (lane 3) and GFP-PSD95 (lane 2) were well expressed. Furthermore, His-nNOS and GFP-PSD95 eluted from PSD95-nNOS/Fe-MOF (lane 4) could be detected at the same position as PSD95-nNOS (lane 1), which further proved that His-nNOS and GFP-PSD95 were successfully immobilized on the magnetic Fe-MOF.

### Stability of PSD95-nNOS/Fe-MOF

**Fig. 5:**
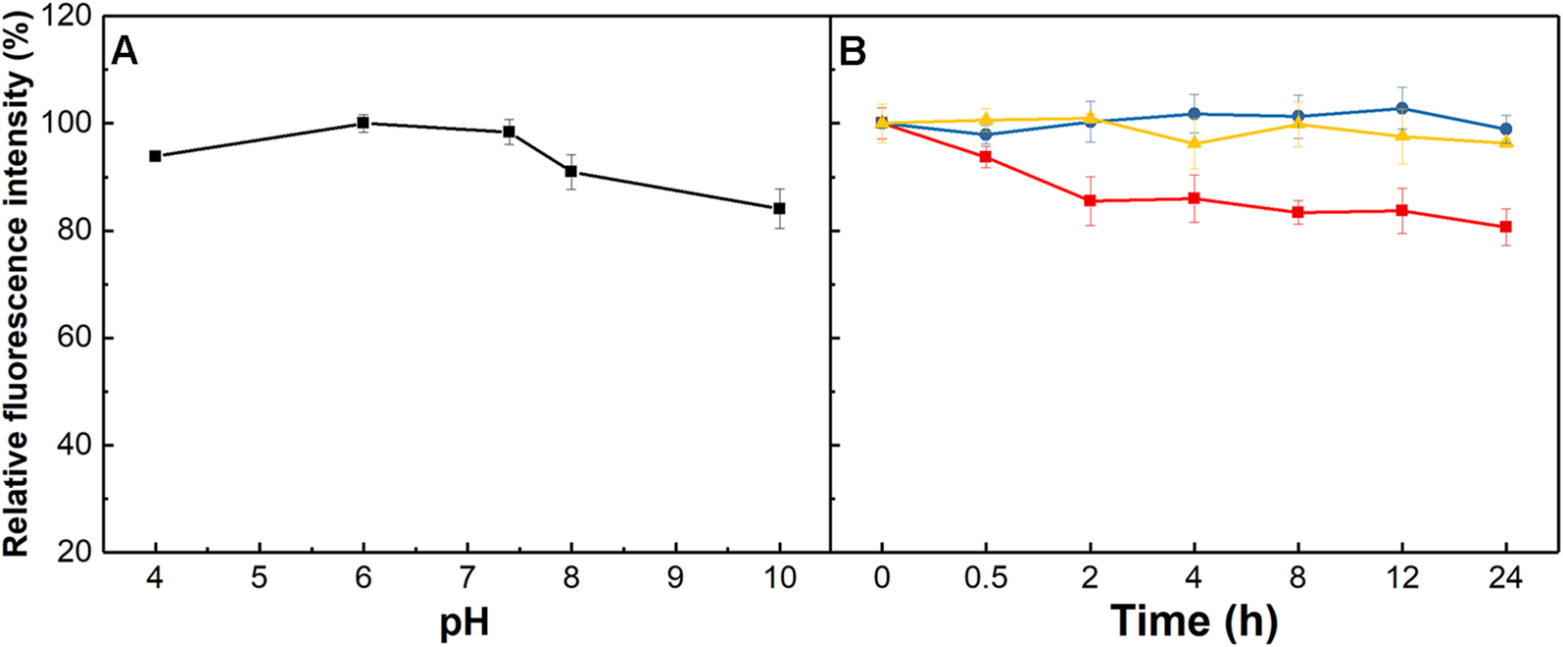
**A** pH stability of PSD95-nNOS/Fe-MOF, **B** time stability of (yellow) PSD95-nNOS/Fe-MOF and (red) free PSD95-nNOS; (blue) PSD95-nNOS/Fe-MOF in 0.1% DMSO

The fluorescence intensity of PSD95-nNOS/Fe-MOF was measured at pH 4, 6, 7.4, 8 and 10 in Fig. 5A. The fluorescence intensity reached the maximum at pH 6 and decreased slightly at pH 7.4. At pH 8 and pH 10, the fluorescence intensity decreased gradually. And the disintegration of magnetic Fe-MOF could be observed at pH 10, which may lead to the detachment of PSD95-nNOS. Although the fluorescence intensity of PSD95-nNOS/Fe-MOF was the highest at pH 6, there was no significant difference between pH 6 and pH 7.4 (P > 0.05). Considering that the proteins are more stable at pH 7.4, therefore, PBS buffer solution (pH 7.4) was chosen as the optimal solution condition to screen the candidates when PSD95-nNOS/Fe-MOF was used as the screening model.

The time stability and solvent stability were described in Fig. 5B. After 24 h, the fluorescence intensity of free PSD95-nNOS lost up to about 20%, nevertheless, PSD95-nNOS immobilized on magnetic Fe-MOF could keep fluorescence intensity almost unchanged. The results indicated that magnetic Fe-MOF could protect the immobilized protein and enhance the stability of PSD95-nNOS.

In addition, PBS wasn’t a good solvent for all candidates, so DMSO (less than 0.1%) was added as a cosolvent to increase some compounds solubility. Therefore, the fluorescence stability of PSD95-nNOS/Fe-MOF in 0.1% DMSO need to be determined. It was exciting that PSD95-nNOS/Fe-MOF in 0.1% DMSO was very stable for 24 h, that is, the addition of 0.1% DMSO would not interfere with the fluorescence intensity of PSD95-nNOS/Fe-MOF (Fig. 5B).

### Anti-interference ability of PSD95-nNOS/Fe-MOF

**Fig. 6:**
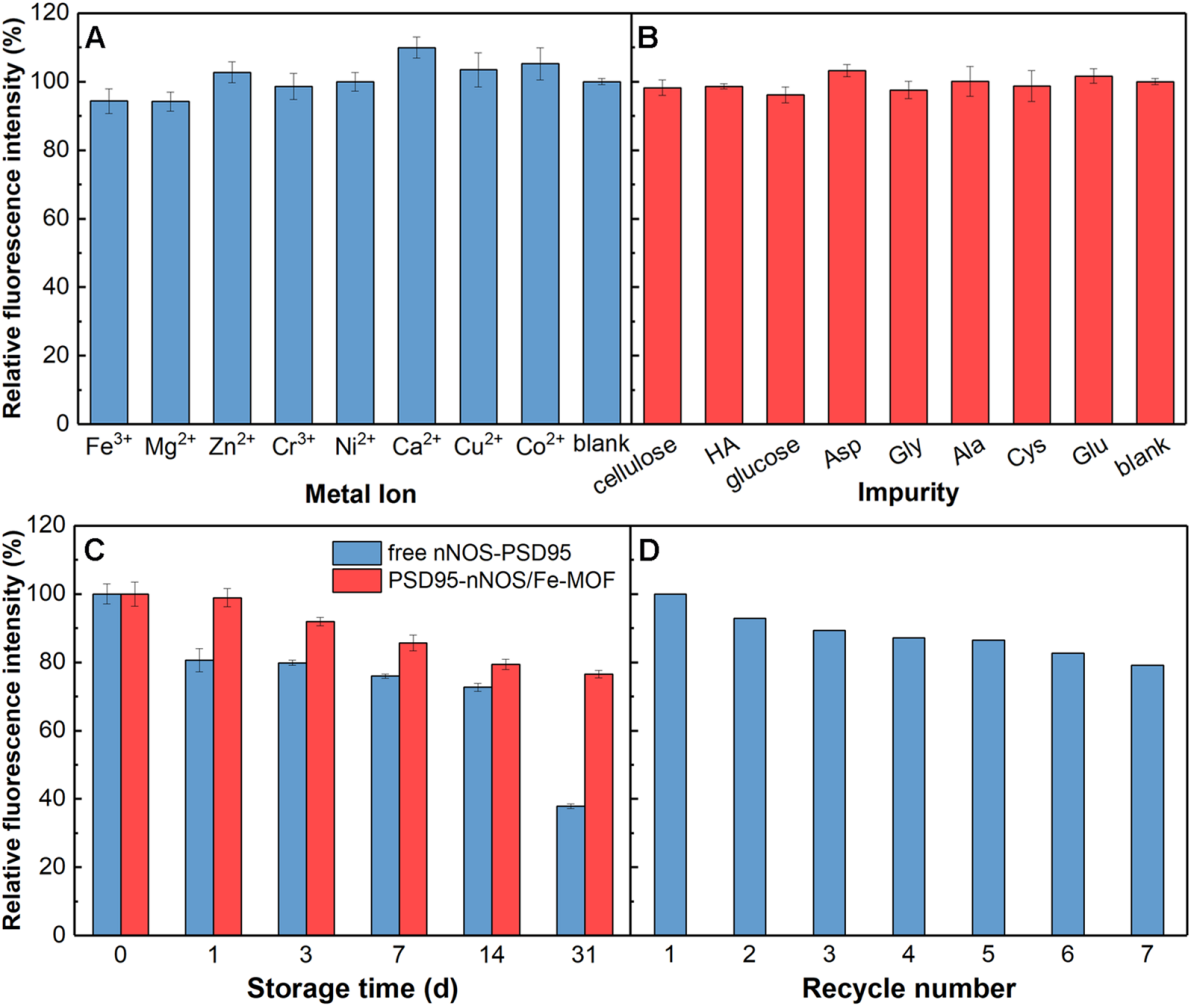
Anti-interference ability of PSD95-nNOS/Fe-MOF to (**A**) metal ion and (**B**) impurities in natural medicine; (**C**) storage stability of free PSD95-nNOS and PSD95-nNOS/Fe-MOF; (**D**) reusability of nNOS/Fe-MOF

In order to quickly discover PSD95-nNOS uncouplers from traditional Chinese medicine and other complex systems, PSD95-nNOS/Fe-MOF is required to have good anti-interference performance. Some impurities such as metal ions (Fe^3+^, Mg^2+^, Zn^2+^, Cr^3+^, Ni^2+^, Ca^2+^, Cu^2+^, Co^2+^), cellulose, hyaluronic acid (HA), glucose and amino acids exist in the extract of the active ingredients from traditional Chinese medicine, so we chose these substances to measure the anti-interference performance of PSD95-nNOS/Fe-MOF. The anti-interference ability of PSD95-nNOS/Fe-MOF was examined through adding 20 μM different impurities, as shown in Fig. 6A, B. The results showed that the fluorescence intensity of each sample was close to the blank sample, which demonstrated the good anti-interference ability of PSD95-nNOS/Fe-MOF.

### Storage stability of PSD95-nNOS/Fe-MOF

The storage stability of PSD95-nNOS/Fe-MOF was also evaluated. After being stored for one day at 4 °C, the relative fluorescence intensity of PSD95-nNOS/Fe-MOF and free PSD95-nNOS were 98.9% and 80.6%, respectively. Thus it can be seen that the loss of fluorescence intensity of PSD95-nNOS/Fe-MOF was very low after one day. In our study, PSD95-nNOS/Fe-MOF nanoparticles weren’t stored for more than 1 day, we always prepared them with an appropriate amount and used up them in time.

After a 3 days incubation, PSD95 in PSD95-nNOS/Fe-MOF suspension or free PSD95-nNOS could remain 91.9% and 79.8%, respectively. After being stored for a month, PSD95-nNOS/Fe-MOF could still maintain 74.71% of the original fluorescence intensity, while free PSD95-nNOS could only keep 37.84% (Fig. 6C). As expected, PSD95-nNOS/Fe-MOF could maintain better storage stability than free PSD95-nNOS.

### Reusability of nNOS/Fe-MOF

To make PSD95-nNOS/Fe-MOF be used as an effective screening system, it is important to investigate the reusability of the immobilized core, that is, nNOS/Fe-MOF, after all, more usage means more convenient operations and less cost. Reusability of nNOS/Fe-MOF was inspected by coupling with GFP-PSD95 and then uncoupling with ZL006. Figure 6D shows that the fluorescence intensity of PSD95-nNOS/Fe-MOF could still maintain 80% after 7 cycles, which further verified the protective effect of magnetic Fe-MOF on PSD95-nNOS. Compared with the other methods in Table 2, PSD95 could be rapidly and quantitatively detected in this work, which was very significant for discovering PSD95-nNOS uncouplers by high-throughput screening.

**Table 2:** Comparison of the methods to detect PSD95

Detection system	PSD95-nNOS	PSD95/Discs large/ZO-1	b1AR-PSD95	PSD95-nNOS/Fe-MOF
Method	Western blotting	NMR spectroscopy	Fluorescence imaging	Fluorescence detection
Main advantages	High sensitivity, high selectivity	Detection of conformational changes	Enhancement of detection signal	Low cost, high sensitivity, high-throughput screening
Limitation	Only qualitative analysis	Difficulty in high-throughput screening		Limited recycle number
Reference	[42]	[43]	[44]	This work

### Validation of the effectiveness of the screening model in vitro using ZL006

**Fig. 7:**
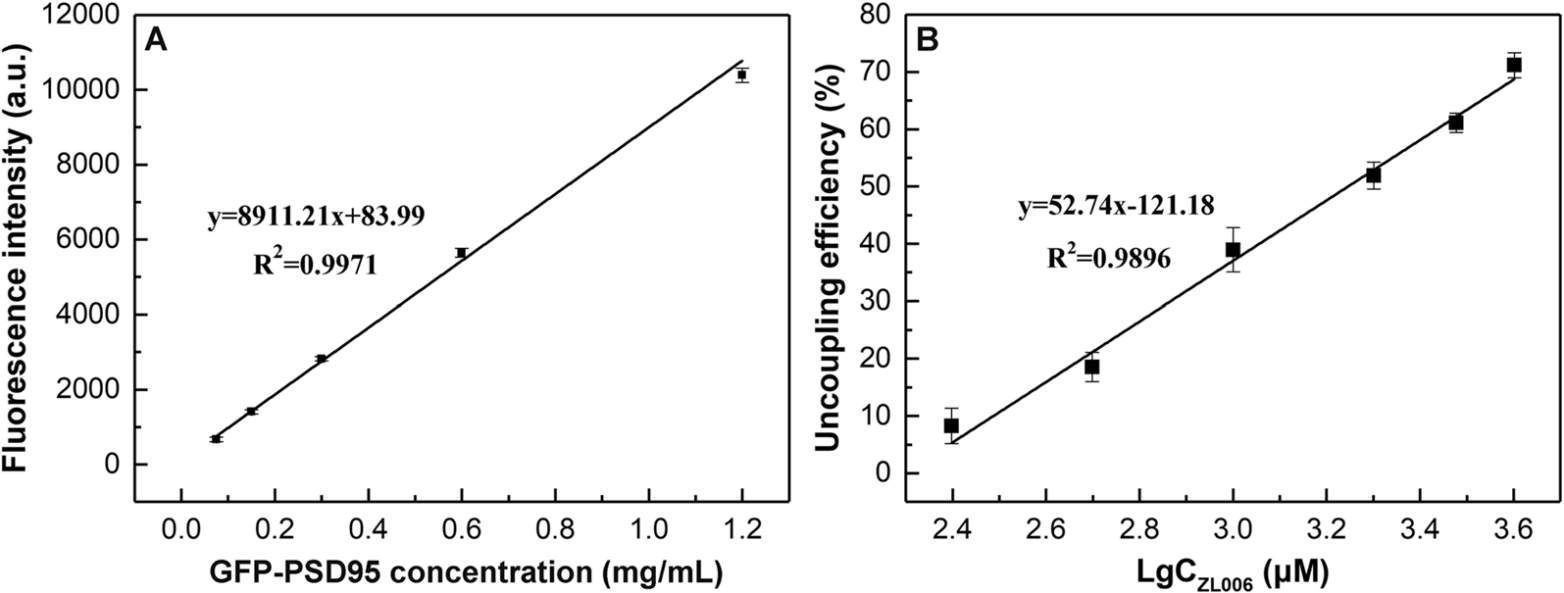
The linear plot of the fluorescence intensity versus GFP-PSD95 concentration (**A**). The uncoupling efficiency versus LgC_ZL006_ (**B**)

It was proved that the fluorescence intensity and the concentration of GFP-PSD95 solution had a good linear correlation in the range of 0.075–1.2 mg/mL (Fig. 7A). Based on the experiment of testifying the feasibility of PSD95-nNOS/Fe-MOF screening system using ZL006, the suspension was magnetically separated and the fluorescence intensity of the precipitate was measured to analyze the uncoupling efficiency after ZL006 was incubated with PSD95-nNOS/Fe-MOF. The uncoupling efficiency and logarithm of the concentration of ZL006 showed a good linear relationship in the range of 250–4000 μM, as demonstrated in Fig. 7B. The fitted linear equation was y = 52.74x − 121.18 (R^2^ = 0.9896). The limit of detection (3σ/K, where σ represents the standard deviation of blank signal (n = 10), and K is the slope of the calibration curve) was estimated to be 1.25 µM. From the above results, we concluded that PSD95-nNOS/Fe-MOF could be used as a screening model to seek PSD95-nNOS uncouplers in vitro.

### Discovery of the potential PSD95-nNOS uncouplers

**Fig. 8:**
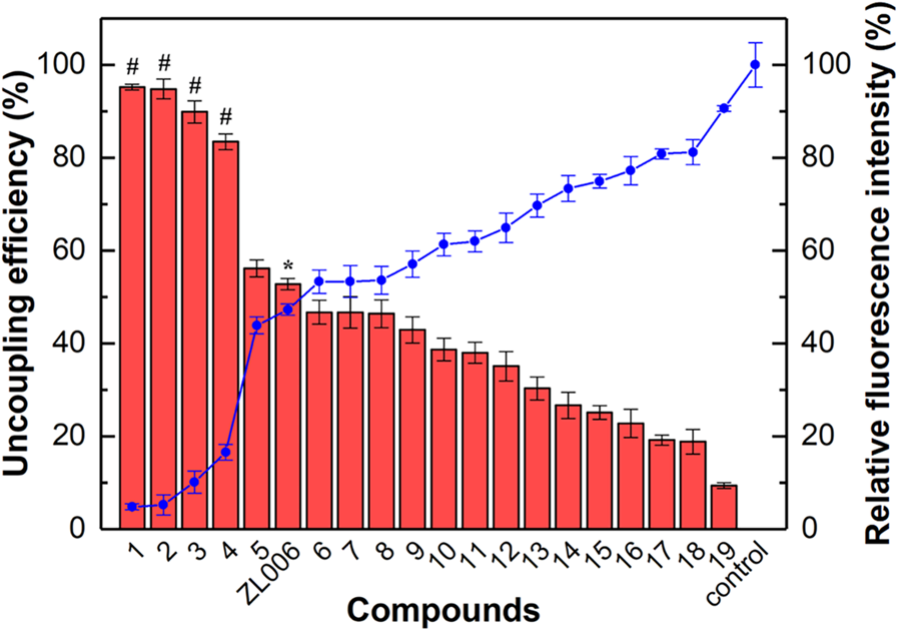
Uncoupling efficiency of the candidates at 1 mM. *P < 0.05, compared with control. #P < 0.05, compared with ZL006.The compounds are: (1) Baicalin, (2) Baicalein, (3) Gnetol, (4) Emodin-8-O-β-D-glucopyranoside, (5) Corylifol A, (6) Isorhapontigenin, (7) Nitidine chloride, (8) Chelerythrine, (9) Physcion, (10) Wogonoside, (11) Resveratrol, (12) Dehydrocorydaline, (13) Hesperidin, (14) Rhaponiticin, (15) Polydatin, (16) Sanguinarine, (17) Neobavaisoflavone, (18) Allodryptopine, (19) Biochanin A

Although PSD95-nNOS/Fe-MOF as the in vitro screening model is effective for ZL006, it need be testified whether it could screen out some potential PSD95-nNOS uncouplers from the candidates, for example, the active ingredients in natural medicine. Nineteen compounds were pre-screened and chosen as the candidates (Additional file 1: Fig. S2), and their uncoupling efficiency was calculated according to the Eq. (2), as described in Fig. 8. After PSD95-nNOS/Fe-MOF particles were incubated with these candidates under certain conditions, we separated the suspensions by magnet and measured the fluorescence intensity of the precipitates. The stronger fluorescence intensity implies the more PSD95 coupled and thereby the weaker of uncoupling efficiency, which was demonstrated by the inverse relationship between fluorescence intensity and uncoupling efficiency in Fig. 8.

The uncoupling efficiencies of Baicalin, Baicalein, Emodin-8-*O*-β-d-glucopyranoside and Gnetol were very outstanding, all of which were better than that of ZL006 as the positive control. The other compounds including Corylifol A, Nitidine chloride, Chelerythrine, Isorhapontigenin and Physcion, whose uncoupling efficiencies were similar with ZL006’s, also could be designed as the potential uncouplers. As expected, the above known uncouplers had been screened out, which further demonstrated PSD95-nNOS/Fe-MOF was a good system to screen PSD95-nNOS uncouplers in vitro.

IC_50_ values of these compounds were subsequently determined. Herein, IC_50_ value was defined as the half-maximal inhibition concentration against PSD95-nNOS coupling, consequently, the lower IC_50_ signifies the better uncoupling ability of the compound. All IC_50_ of Baicalin, Baicalein, Emodin-8-O-β-D-glucopyranoside, Gnetol and Corylifol A were less than ZL006, which was consistent with the results in Fig. 8 and reflected the satisfactory sensitivity and accuracy of this screening method (Table 3).

**Table 3:** Inhibition effects of candidates evaluated by IC_50_

Compounds	Structure	IC_50_ (mM)
ZL006		1.805 ± 0.151
Baicalin		0.104 ± 0.001
Baicalein		0.144 ± 0.022
Emodin-8-*O*-β-d-glucopyranoside		0.505 ± 0.082
Gnetol		0.492 ± 0.063
Corylifol A		1.801 ± 0.034
Nitidine chloride		> 1.805
Chelerythrine		> 1.805
Isorhapontigenin		> 1.805
Physcion		> 1.805

The data reported represent the means ± SD (n = 3)

## Conclusion

In summary, we first proposed a novel method based on magnetic Fe-MOF for rapidly and sensitively screening of PSD95-nNOS uncouplers. The new PSD95-nNOS/Fe-MOF nanoparticles were fabricated via layer-by-layer self-assembly and systematically characterized, which were constructed by firstly immobilizing His-nNOS on magnetic Fe-MOF, and then interacting with GFP-PSD95. The MMOF nanoparticles were evaluated by fluorescence intensity and showed great stability, anti-interference ability and recyclability, which greatly enhanced the practicality of the system. Furthermore, the system was applied to screen the candidates, Baicalin, Baicalein, Emodin-8-*O*-β-d-glucopyranoside, Gnetol and Corylifol A were finally screened as potential PSD95-nNOS uncouplers and are being used for further study. Therefore, we put forward a new strategy through fabricating multifunctional PSD95-nNOS/Fe-MOF nanoparticles as an effective screening model in vitro for efficiently discovering the potential PSD95-nNOS uncouplers.

## Methods

### Materials

Ferric chloride hexahydrate (FeCl_3_·6H_2_O), ammonium hydroxide (NH_3_·H_2_O), acetic acid and Polyethylene glycol 200 (PEG 200) were purchased from Sinopharm Chemical Reagent Co., Ltd. (Shanghai, China). 2-Aminoterephthalic acid and Rhodamine B were purchased from Aladdin (Shanghai, China). Ferrous chloride tetrahydrate (FeCl_2_·4H_2_O) was obtained from Shanghai Richjoint Chemical Reagents Co., Ltd. (Shanghai, China). All the standards were purchased from Nanjing Yuanbaofeng Pharm-Tech Co., Ltd. (Nanjing, China). 4-(3,5-Dichloro-2-hydroxy-benzylamino)-2-hydroxybenzoic acid (ZL006) was kindly offered by the Department of medicinal chemistry, Nanjing Medical University (Nanjing, China). The Bradford Protein Assay Kit was purchased from Cwbiotech Co., Ltd. (Taizhou, China). The recombinant plasmids pcDNA3.1-myc-His-nNOS, pcDNA3.1-Flag-nNOS and pcDNA3.1-GFP-PSD95-Flag were all constructed by Geneppl technology Co., Ltd. (Nanjing, China).

### Acquisition of nNOS and GFP-PSD95 proteins

Neuronal nitric oxide synthase from *Rattus norvegicus* (nNOS, gene ID: 24598) and postsynaptic density protein-95 from *Homo sapiens* (PSD95, gene ID: 1742) were total synthesized. The recombinant plasmids pcDNA3.1-myc-His-nNOS, pcDNA3.1-Flag-nNOS and pcDNA3.1-GFP-PSD95-Flag were transfected into human embryonal kidney (HEK) 293 T cells using the transfection reagent Lipo293™, respectively. Transfected cells were cultured in DMEM medium (37 °C, 5% CO_2_) for another 48 h and collected by centrifugation and washed with cold phosphate buffer solution three times, then lysed with RIPA lysis buffer. Finally, the lysates were centrifuged at 12,000*g* for 15 min to remove cell debris and gain the crude protein solutions. The proteins His-nNOS, Flag-nNOS and GFP-PSD95-Flag (Hereinafter referred to as GFP-PSD95) were verified by western blotting (Additional file 1: Fig. S1).

### Synthesis of magnetic Fe_3_O_4_ particles

Fe_3_O_4_ particles were synthesized based on the reported procedures [14]. In brief, 10 mmol FeCl_2_·4H_2_O and 20 mmol FeCl_3_·6H_2_O were dissolved in 80 mL ultrapure water. The mixture was stirred intensely for 3 min and purged with nitrogen gas while the temperature increased to 80 °C. Then 10 mL NH_3_·H_2_O was added and stirred for 1 h, mixed with 2 mL PEG 200 and continuously stirred for 10 min. The products were magnetically separated, washed several times with water and then dried in vacuum for further use.

### The layer-by-layer self-assembly process

In the synthesis of the magnetic Fe-MOF layer [29, 45], 0.5 g 2-aminoterephthalic acid was dissolved in 60 mL *N*,*N*-dimethylformamide, followed by adding 0.6 g Fe_3_O_4_ particles. The mixture was stirred at 120 °C, 175 rpm for 4 h, then mixed with 800 μL acetic acid which was preheated for 15 min. After slowly cooling down, the products were magnetically separated, washed with 20 mL *N*,*N*-dimethylformamide and 20 mL ethanol, and dried in vacuum for further use.

Typically, magnetic Fe-MOF (3 mg) was uniformly dispersed in 250 μL His-nNOS phosphate buffer solution (PBS) (1 mg/mL, pH 7.4), then stirred at 4 °C, 175 rpm for 75 min to obtain His-nNOS layer on Fe-MOF by coordinative combination, the nanoparticles obtained in this step were named as His-nNOS/Fe-MOF. The products were magnetically separated and washed with PBS once, and stored at 4 °C for later use. Similarly, Flag-nNOS/Fe-MOF particles as control were prepared like His-nNOS/Fe-MOF just replacing His-nNOS with Flag-nNOS.

The products were magnetically separated and the proteins in the solution were determined via Bradford method. The loading efficiency was calculated using the following Eq. (1):

1 $$\mathrm{Loading efficiency \%}=\frac{{C}_{0}-{C}_{t}}{{C}_{0}}\times 100\%,$$

where *C*_*0*_ and *C*_*t*_ are the concentrations of His-nNOS in the initial solution before immobilization and the solution after immobilization for t minutes, respectively.

PSD95 layer on His-nNOS/Fe-MOF particles was prepared according to the reported procedures with minor modifications [34]. 3.6 mg His-nNOS/Fe-MOF were dispersed in 300 μL GFP-PSD95 PBS buffer (1.25 mg/mL) and shaken at 4 °C, 175 rpm overnight to obtain multilayer nanoparticles, namely PSD95-nNOS/Fe-MOF. The biocomposites were magnetically separated and stored at 4 °C until further use. In brief, PSD95-nNOS/Fe-MOF multilayer nanoparticles were step by step prepared, as described in Scheme 1.

### Characterization

The morphologies were observed by scanning electron microscopy (SEM) using a Quanta 400 FEG (USA) and transmission electron microscopy (TEM) using a JEM 2100F (Japan). The Fourier transform infrared spectroscopy (FT-IR), X-ray diffraction (XRD) and Brunauer–Emmett–Teller (BET) specific surface area were recorded by a Nicolet iS20 (USA), a smartlab9 (Japan) and a TriStar II 3020 (USA), respectively. The confocal laser scanning microscopy images were obtained from a LSM 800 (German). The magnetic performance was examined by a PPMS-9T (USA). The thermal gravimetric analysis was recorded by a 209 F3 Tarsus^®^ (German). The circular dichroism spectra were obtained from a Chirascan (UK). The fluorescence intensity was detected by a dual-mode microplate reader (Infinite M NANO^+^, Switzerland).

The detailed description of characterization was shown in the “Additional file 1”.

### Stability of PSD95-nNOS/Fe-MOF

To evaluate the stability of PSD95-nNOS/Fe-MOF, 300 μL His-nNOS PBS buffer, or 3.6 mg His-nNOS/Fe-MOF (with the same protein amount at 0.225 mg) were mixed with 300 μL GFP-PSD95 PBS buffer (1.25 mg/mL) and shaken at 4 °C, 175 rpm overnight to obtain free PSD95-nNOS and PSD95-nNOS/Fe-MOF, respectively. Then, 1.8 mg PSD95-nNOS/Fe-MOF were separately dispersed in 300 µL PBS buffer and 300 µL PBS buffer with 0.1% DMSO. The free PSD95-nNOS solution, PSD95-nNOS/Fe-MOF PBS suspension, PSD95-nNOS/Fe-MOF PBS suspension with 0.1% DMSO were left standing at 4 °C for 0, 0.5, 2, 4, 8, 12 and 24 h, respectively. The fluorescence intensities of the coupled GFP-PSD95 were all measured.

The pH stability was also evaluated. Typically, 1.8 mg PSD95-nNOS/Fe-MOF were added in 300 µL PBS buffer of various pH (4, 6, 7.4, 8 and 10), respectively. The evenly dispersed mixture was shaken at 4 °C, 175 rpm for 5 h. The supernatant was removed by magnetic separation and the fluorescence intensities of PSD95-nNOS/Fe-MOF were measured. All trials above were performed in triplicate.

### Anti-interference performance of PSD95-nNOS/Fe-MOF

1.8 mg PSD95-nNOS/Fe-MOF were dispersed in different impurities (300 µL, 20 µM) and shaken at 4 °C, 175 rpm for 5 h, respectively. The suspension was magnetically separated and the fluorescence intensities of the precipitates were measured. All trials were performed in triplicate.

### Storage stability of PSD95-nNOS/Fe-MOF

300 µL free PSD95-nNOS and 300 µL PSD95-nNOS/Fe-MOF PBS buffer were left standing at 4 °C for 1, 3, 7, 14, and 31 days, and the fluorescence intensities of the coupled GFP-PSD95 were measured.

### Reusability of nNOS/Fe-MOF

3.6 mg PSD95-nNOS/Fe-MOF were uncoupled with 600 μL of 4 mM ZL006 PBS buffer at 4 °C for 5 h (125 rpm), then the solution was magnetically separated and the precipitate was recombined with PSD95 to obtain PSD95-nNOS/Fe-MOF by adding 300 μL GFP-PSD95 solution (1.25 mg/mL) and shaking at 4 °C overnight (175 rpm). The regained PSD95-nNOS/Fe-MOF was separated by magnet and uncoupled by ZL006 again. This procedure was repeated 7 times, and the fluorescence intensity of PSD95-nNOS/Fe-MOF was measured each time.

### Fluorescence intensity measurements

The GFP-PSD95 solutions were serially diluted with PBS buffer to make test samples with final concentrations of 1.2, 0.6, 0.3, 0.15 and 0.075 mg/ml. The fluorescence intensities of GFP-PSD95 with different concentrations were recorded at 506 nm with the excitation wavelength of 475 nm. All trials were performed in triplicate.

### Application of PSD95-nNOS/Fe-MOF to discover potential PSD95-nNOS uncouplers

We chose ZL006, a known PSD95-nNOS uncoupler [10], to further testify the feasibility of the system. 1.8 mg PSD95-nNOS/Fe-MOF and 300 μL different concentrations of ZL006 (0, 0.5, 1, 2, 3 and 4 mM) were incubated at 4 °C, 125 rpm for 5 h. Then the suspension was magnetically separated and the fluorescence intensity of the precipitate was measured, respectively. The uncoupling efficiency was calculated as Eq. (2):

2 $$\mathrm{Uncoupling efficiency \%}=\left(1-\frac{{F}_{c}-{F}_{Blank}}{{F}_{0}-{F}_{Blank}}\right)\times 100\mathrm{\%},$$

where *F*_*0*_ and *F*_*C*_ are the fluorescence intensities of the coupled PSD95 on PSD95-nNOS/Fe-MOF interacting with ZL006 at 0 mM and the other concentrations respectively, *F*_*Blank*_ denotes the fluorescence intensity of the solvent.

The screened compounds (1 mM) were separately dissolved by 300 μL PBS buffer with 0.1% DMSO, and the following addition of 1.8 mg PSD95-nNOS/Fe-MOF was carried out. The suspension was mixed well and added to a 96-well plate, and shaken at 4 °C for 5 h (125 rpm). Then PSD95-nNOS/Fe-MOF were magnetically separated and the fluorescence intensities were measured. The uncoupling efficiency was calculated according to Eq. (2). After comparing the effects of the candidates on dissociating GFP-PSD95, we chose some outstanding hits to assay their IC_50_.

## Supplementary Information

**Additional file 1**: Preparation of the contrasting materials; optimization of His-nNOS immobilization conditions; characterization; western blot analysis; the pre-screening experiments on the compounds. **Fig. S1**. Western blotting analysis (M: marker, lane 1: PSD95-nNOS, lane 2: GFP-PSD95, lane 3: His-nNOS, lane 4: PSD95-nNOS/Fe-MOF, the lanes on the left of the marker were detected with nNOS-specific antibodies and the lanes on the right of the marker were detected with PSD95-specific antibodies). **Fig. S2**. The quenching effect of nineteen compounds on the fluorescence intensity of GFP-PSD95. **Table S1**. Comparison of the particle size for the materials to immobilize nNOS.

## Data Availability

All data used to support the conclusions of this study are included within the article and its Additional files.

## Abbreviations

PSD95: Postsynaptic density protein-95
nNOS: Neuronal nitric oxide synthase
MOF: Metal–organic framework
GFP: Green fluorescent protein
RFP: Red fluorescent protein
YFP: Yellow fluorescent protein
NMDAR: *N*-Methyl-d-aspartate receptor
MCAO/R: Middle cerebral artery occlusion and reperfusion
ELISA: Enzyme-linked immunosorbent assay
FeCl_3_·6H_2_O: Ferric chloride hexahydrate
NH_3_·H_2_O: Ammonium hydroxide
PEG 200: Polyethylene glycol 200
RhB: Rhodamine B
FeCl_2_·4H_2_O: Ferrous chloride tetrahydrate
SBA-15: Santa Barbara Amorphous-15
DMEM: Dulbecco’s Modified Eagle Medium
RIPA lysis buffer: Radioimmunoprecipitation assay lysis buffer
PBS: Phosphate buffer solution
HA: Hyaluronic acid
ZL006: 4-(3,5-Dichloro-2-hydroxy-benzylamino)-2-hydroxybenzoic acid
SEM: Scanning electron microscopy
TEM: Transmission electron microscopy
FT-IR: Fourier transform infrared spectroscopy
XRD: X-ray diffraction
BET: Brunauer–Emmett–Teller
DMSO: Dimethyl sulfoxide
IC_50_: Half-maximal inhibitory concentration

## References

[1] Khoshnam SE, Winlow W, Farzaneh M, Farbood Y, Moghaddam HF (2017). Pathogenic mechanisms following ischemic stroke. Neurol Sci.

[2] Ehresman J, Cottrill E, Caplan JM, McDougall CG, Theodore N, Nyquist PA (2021). Neuroprotective role of acidosis in ischemia: review of the preclinical evidence. Mol Neurobiol.

[3] Hao YL, Xin MY, Feng LS, Wang XY, Wang X, Ma D, Feng JC (2020). Review cerebral ischemic tolerance and preconditioning: methods, mechanisms, clinical applications, and challenges. Front Neurol.

[4] Barthels D, Das H (2020). Current advances in ischemic stroke research and therapies. Biochim Biophys Acta BBA Mol Basis Dis..

[5] Wendelboe AM, Raskob GE (2016). Global burden of thrombosis epidemiologic aspects. Circ Res.

[6] Ge Y, Chen WL, Axerio-Cilies P, Wang YT (2020). NMDARs in cell survival and death: implications in stroke pathogenesis and treatment. Trends Mol Med.

[7] Lai TW, Zhang S, Wang YT (2014). Excitotoxicity and stroke: identifying novel targets for neuroprotection. Prog Neurobiol.

[8] Qu W, Liu NK, Wu X, Wang Y, Xia Y, Sun Y, Lai Y, Li R, Shekhar A, Xu XM (2020). Disrupting nNOS–PSD95 interaction improves neurological and cognitive recoveries after traumatic brain injury. Cereb Cortex.

[9] Wang Z, Chen Z, Yang J, Yang Z, Yin J, Duan X, Shen H, Li H, Wang Z, Chen G (2019). Treatment of secondary brain injury by perturbing postsynaptic density protein-95-NMDA receptor interaction after intracerebral hemorrhage in rats. J Cereb Blood Flow Metab..

[10] Zhou L, Li F, Xu HB, Luo CX, Wu HY, Zhu MM, Lu W, Ji X, Zhou QG, Zhu DY (2010). Treatment of cerebral ischemia by disrupting ischemia-induced interaction of nNOS with PSD-95. Nat Med.

[11] Cai W, Wu S, Pan Z, Xiao J, Li F, Cao J, Zang W, Tao YX (2018). Disrupting interaction of PSD-95 with nNOS attenuates hemorrhage-induced thalamic pain. Neuropharmacology.

[12] David J, O'Toole E, O'Reilly K, Thuery G, Assmann N, Finlay D, Harkin A (2018). Inhibitors of the NMDA-nitric oxide signaling pathway protect against neuronal atrophy and synapse loss provoked by l-alpha aminoadipic acid-treated astrocytes. Neuroscience.

[13] Chen D, Zhao T, Ni K, Dai P, Yang L, Xu Y, Li F (2016). Metabolic investigation on ZL006 for the discovery of a potent prodrug for the treatment of cerebral ischemia. Bioorg Med Chem Lett.

[14] Yao D, Zhang L, Huang J, Sun C, Zhang Y, Gu X, Wang CZ, Li F, Chen L, Yuan CS (2018). A surface magnetic imprinted polymers as artificial receptors for selective and efficient capturing of new neuronal nitric oxide synthase-post synaptic density protein-95 uncouplers. J Pharm Biomed Anal.

[15] Huang J, Sun C, Yao D, Wang CZ, Zhang L, Zhang Y, Chen L, Yuan CS (2018). Novel surface imprinted magnetic mesoporous silica as artificial antibodies for efficient discovery and capture of candidate nNOS-PSD-95 uncouplers for stroke treatment. J Mater Chem B.

[16] Pan LL, Ding YY, Ni XT, Wang CZ, Jiang B, Zhang Y, Jiang N, Tang YL, Chen LN, Yuan CS (2020). Modeling rapid and selective capture of nNOS-PSD-95 uncouplers from Sanhuang Xiexin decoction by novel molecularly imprinted polymers based on metal-organic frameworks. RSC Adv.

[17] Liang S, Wu XL, Xiong J, Zong MH, Lou WY (2020). Metal-organic frameworks as novel matrices for efficient enzyme immobilization: an update review. Coord Chem Rev.

[18] Lian X, Fang Y, Joseph E, Wang Q, Li J, Banerjee S, Lollar C, Wang X, Zhou HC (2017). Enzyme-MOF (metal-organic framework) composites. Chem Soc Rev.

[19] Liang W, Wied P, Carraro F, Sumby CJ, Nidetzky B, Tsung CK, Falcaro P, Doonan CJ (2021). Metal-organic framework-based enzyme biocomposites. Chem Rev.

[20] Zhang A, Zong H, Fu H, Wang L, Cao X, Zhong Y, Liu B, Liu J (2022). Controllable synthesis of nickel doped hierarchical zinc MOF with tunable morphologies for enhanced supercapability. J Colloid Interface Sci.

[21] Huang X, Zhang S, Tang Y, Zhang X, Bai Y, Pang H (2021). Advances in metal–organic framework-based nanozymes and their applications. Coord Chem Rev.

[22] Freund R, Lächelt U, Gruber T, Rühle B, Wuttke S (2018). Multifunctional efficiency: extending the concept of atom economy to functional nanomaterials. ACS Nano.

[23] Man T, Xu C, Liu XY, Li D, Tsung CK, Pei H, Wan Y, Li L (2022). Hierarchically encapsulating enzymes with multi-shelled metal-organic frameworks for tandem biocatalytic reactions. Nat Commun.

[24] Hsu PH, Chang CC, Wang TH, Lam PK, Wei MY, Chen CT, Chen CY, Chou LY, Shieh FK (2021). Rapid fabrication of biocomposites by encapsulating enzymes into Zn-MOF-74 via a mild water-based approach. ACS Appl Mater Interfaces.

[25] Neupane S, Patnode K, Li H, Baryeh K, Liu G, Hu J, Chen B, Pan Y, Yang Z (2019). Enhancing enzyme immobilization on carbon nanotubes via metal-organic frameworks for large-substrate biocatalysis. ACS Appl Mater Interfaces.

[26] Luo J, Dai Y, Xu X, Liu Y, Yang S, He H, Sun C, Xian Q (2022). Green and efficient synthesis of Co-MOF-based/g-C3N4 composite catalysts to activate peroxymonosulfate for degradation of the antidepressant venlafaxine. J Colloid Interface Sci.

[27] Sassolas A, Blum LJ, Leca-Bouvier BD (2012). Immobilization strategies to develop enzymatic biosensors. Biotechnol Adv.

[28] Roeder R, Preiss T, Hirschle P, Steinborn B, Zimpel A, Hoehn M, Raedler JO, Bein T, Wagner E, Wuttke S, Laechelt U (2017). Multifunctional nanoparticles by coordinative self-assembly of His tagged units with metal organic frameworks. J Am Chem Soc.

[29] Shi S, Zhang W, Wu H, Li Y, Ren X, Li M, Liu J, Sun J, Yue T, Wang J (2020). In situ cascade derivation toward a hierarchical layered double hydroxide magnetic absorbent for high-performance protein separation. ACS Sustain Chem Eng.

[30] Niu H, Ding M, Sun X, Zhuang W, Liu D, Ying H, Zhu C, Chen Y (2019). Immobilization of a polyphosphate kinase 2 by coordinative self-assembly of His-tagged units with metal-organic frameworks and its application in ATP regeneration from AMP. Colloids Surf B.

[31] Jin X, Ye Q, Wang CW, Wu Y, Ma K, Yu S, Wei N, Gao H (2021). Magnetic nanoplatforms for covalent protein immobilization based on spy chemistry. ACS Appl Mater Interfaces.

[32] Cohen L, Walt DR (2019). Highly sensitive and multiplexed protein measurements. Chem Rev.

[33] Bleicher AV, Unger HW, Rogerson SJ, Aitken EH (2018). A sandwich enzyme-linked immunosorbent assay for the quantitation of human plasma ferritin. MethodsX.

[34] Mo SF, Liao GY, Yang J, Wang MY, Hu Y, Lian GN, Kong LD, Zhao Y (2016). Protection of neuronal cells from excitotoxicity by disrupting nNOS–PSD95 interaction with a small molecule SCR-4026. Brain Res.

[35] Gallo E (2020). Fluorogen-activating proteins: next-generation fluorescence probes for biological research. Bioconjug Chem.

[36] Cai L, Li H, Yu X, Wu L, Wei X, James TD, Huang C (2021). Green fluorescent protein GFP-chromophore-based probe for the detection of mitochondrial viscosity in living cells. ACS Appl Bio Mater.

[37] Fang X, Zheng Y, Duan Y, Liu Y, Zhong W (2019). Recent advances in design of fluorescence-based assays for high-throughput screening. Anal Chem.

[38] He H, Gu X, Shi L, Hong J, Zhang H, Gao Y, Du S, Chen L (2015). Molecularly imprinted polymers based on SBA-15 for selective solid-phase extraction of baicalein from plasma samples. Anal Bioanal Chem.

[39] Mahmoodi NM, Abdi J (2019). Metal-organic framework as a platform of the enzyme to prepare novel environmentally friendly nanobiocatalyst for degrading pollutant in water. J Ind Eng Chem.

[40] Hirst JD, Colella K, Gilbert ATB (2003). Electronic circular dichroism of proteins from first-principles calculations. J Phys Chem B.

[41] Li S, Peng Z, Leblanc RM (2015). Method to determine protein concentration in the protein nanoparticle conjugates aqueous solution using circular dichroism spectroscopy. Anal Chem.

[42] Karadayian AG, Bustamante J, Lores-Arnaiz S (2021). Alcohol hangover induces nitric oxide metabolism changes by impairing NMDA receptor-PSD95-nNOS pathway. Nitric Oxide.

[43] Frederiksen RS, Alarcon-Llado E, Krogstrup P, Bojarskaite L, Buch-Månson N, Bolinsson J, Nygård J, Morral AFI, Martinez KL (2016). Nanowire-aperture probe: local enhanced fluorescence detection for the investigation of live cells at the nanoscale. ACS Photonics.

[44] Zhang J, Sapienza PJ, Ke H, Chang A, Hengel SR, Wang H, Phillips GN, Lee AL (2010). Crystallographic and nuclear magnetic resonance evaluation of the impact of peptide binding to the second PDZ domain of protein tyrosine phosphatase 1E. Biochemistry.

[45] Yin L, Wang Y, Tan R, Li H, Tu Y (2021). Determination of beta-amyloid oligomer using electrochemiluminescent aptasensor with signal enhancement by AuNP/MOF nanocomposite. Microchim Acta.

