# Supplementary material for: Fabrication of multifunctional metal–organic frameworks nanoparticles via layer-by-layer self-assembly to efficiently discover PSD95-nNOS uncouplers for stroke treatment
Source: J Nanobiotechnology. 2022 Aug 13;20:379. doi: 10.1186/s12951-022-01583-7 (PMC9375364; doi:10.1186/s12951-022-01583-7)
Supplement: Supplementary file 1 — Additional file 1: Preparation of the contrasting materials; optimization of His-nNOS immobilization conditions; characterization; western blot analysis; the pre-screening experiments on the compounds. Fig. S1. Western blotting analysis (M: marker, lane 1: PSD95-nNOS, lane 2: GFP-PSD95, lane 3: His-nNOS, lane 4: PSD95-nNOS/Fe-MOF, the lanes on the left of the marker were detected with nNOS-specific antibodies and the lanes on the right of the marker were detected with PSD95-specific antibodies). Fig. S2. The quenching effect of nineteen compounds on the fluorescence intensity of GFP-PSD95. Table S1. Comparison of the particle size for the materials to immobilize nNOS. [file 12951_2022_1583_MOESM1_ESM.doc]

**Supporting Information for**

**Fabrication of multifunctional metal-organic frameworks nanoparticles via layer-by-layer self-assembly to efficiently discover PSD95-nNOS uncouplers for stroke treatment**

Yingying Ding ^1^, Yang Jin ^1^, Tao Peng ^1^, Yankun Gao ^1^, Yang Zang ^2^, Hongliang He ^3^, Fei Li ^1^, Yu Zhang ^1^,*, Hongjuan Zhang ^1^,*, Lina Chen ^1^,*

^1^ School of Pharmacy, Nanjing Medical University, Nanjing, Jiangsu 211166, PR China

^2^ College of Economics & Management, Anhui Agricultural University, Hefei, Anhui 230036, PR China

^3^ Department of Pharmacy, Sir Run Run Hospital, Nanjing Medical University, Nanjing, Jiangsu 211166, PR China

Corresponding Authors

*E-mail: zhangyu@njmu.edu.cn

*E-mail: zhanghj@njmu.edu.cn

*E-mail: chenlina@njmu.edu.cn

**Preparation of the contrasting materials.**

**Preparation of** **Fe-COOH-MOF.**

12 mmol FeCl_3_⋅6H_2_O and 2 mmol sodium citrate were dissolved in 60 mL glycol, then 44 mmol anhydrous sodium acetate was added [1,2]. The solution was continuously stirred for 30 min, then transferred to a Teflon-lined stainless steel autoclave and heated at 200℃ for 12 h. The precipitate was magnetically collected and washed with ethanol for three times. Finally, the product was dried to obtain Fe_3_O_4_-COOH in vacuum at 50 °C for 24 h.

2.8 mmol FeCl_3_∙6H_2_O and 2.8 mmol 2-aminoterephthalic acid (NH_2_-BDC) were suspended in 60 mL DMF, 50 mg Fe_3_O_4_-COOH was added. The mixture was heated at 120 °C for 4 h, then 800 μL acetic acid was added, which was heated for 15 min in advance. After the mixture was slowly cooled down, the precipitate was magnetically collected and washed with N, N-dimethylformamide (DMF) and ethanol for three times. Finally, the product was dried in vacuum at 50 °C for 24 h.

**Preparation of** **UiO-66-NH_2._**

2.8 mmol NH_2_-BDC was dissolved in 20 mL DMF, and 2 mmol ZrCl_4_ was dissolved in 18 mL mixed solution (HCl: DMF, 1:5 V/V) [3]. Then the above solution was mixed in a Teflon-lined stainless steel autoclave and heated at 80 °C overnight. Finally, the precipitate was collected by centrifugation and washed with DMF and ethanol for three times. Finally, the product was dried in vacuum at 50 °C for 24 h.

**Preparation of** **Zr-fum.**

7.2 mmol ZrCl_4_ and 22 mmol fumaric acid were dissolved in solution, which was mixed by 210 mL ultrapure water and 14 mL formic acid [4]. Then the solution was transferred to a Teflon-lined stainless steel autoclave and heated at 120 °C for 24 h. After the mixture was cooled down to room temperature, the precipitate was collected by centrifugation and washed with ultrapure water for three times. Finally, the product was redispersed in ethanol and dried in vacuum at 50 °C for 24 h.

**Preparation of** **HKUST-1.**

3 mmol Cu(NO_3_)_2_·2.5H_2_O was dissolved in 6 mL ultrapure water, and then 6 mmol trimesic acid was added into the solution [4]. The mixture was continuously stirred for 60 min. Then the precipitate was collected by centrifugation and washed with ethanol for three times. Finally, the product was dried in vacuum at 50 °C for 24 h.

**Preparation of** **Cu-BDC.**

1.2 g polyvinyl pyrrolidone was dissolved in mixed solution of 24 mL DMF and 24 mL ethanol, then 0.14 g Cu(NO_3_)_2_·2.5H_2_O and 0.13 g NH_2_-BDC were dissolved in 24 mL DMF [5]. The above solution was mixed in a Teflon-lined stainless steel autoclave and heated at 100 °C for 8 h. The precipitate was collected by centrifugation and washed with ultrapure water for three times. Finally, the product was dried in vacuum at 50 °C for 24 h.

**Preparation of** **SBA-15.**

3 g Pluronic P123 (P123) was added in the solution of 90 g HCl (2 mol/L) and 23 g ultrapure water, then stirred at room temperature until it completely dissolved in the solution [6]. 6.4 g Tetraethyl-orthosilicate was added with dropwise to the homogeneous solution, which was further stirred at 40 °C for 24 h and heated at 100 °C for 24 h. The precipitate was filtered, washed with water, and air-dried at room temperature. And then it was calcinated in flowing air at 550 °C for 5 h, the template P123 was removed and template-free mesoporous silica SBA-15 was obtained.

**Optimization of His-nNOS immobilization conditions.**

**Optimization of the immobilization time.**

3 mg magnetic Fe-MOF was incubated with 250 μL His-nNOS PBS buffer (1 mg/mL) at 4 °C, and the incubation time was simultaneously inspected for 15, 30, 45, 60, 75 and 90 min. The precipitates were separated by magnetism and the proteins in the solution were determined via Bradford method.

**Optimization on the mass ratio of magnetic Fe-MOF to nNOS**.

1, 2, 3, 4 and 5 mg magnetic Fe-MOF were separately incubated with 250 μL His-nNOS PBS buffer (1 mg/mL) or Flag-nNOS PBS buffer (1 mg/mL) at 4 °C,175 rpm for 75 min. The precipitates were magnetically separated and the proteins in the solution were determined via Bradford method.

**Investigation on the nNOS immobilization by different materials.**

3 mg different materials were separately incubated with 250 μL His-nNOS PBS buffer (1 mg/mL) at 4 °C for 75 min. The deposits were magnetically separated or directly centrifuged, and the protein in the solution was determined via Bradford method. All trials above were performed in triplicate.

**Characterization.**

**SEM and TEM observation**.

The samples of magnetic Fe-MOF and His-nNOS/Fe-MOF for scanning electron microscopy (SEM) and transmission electron microscopy (TEM) were prepared according to “Synthesis of magnetic Fe-MOF” and “Coordinative immobilization of nNOS on magnetic Fe-MOF surface”. 3 mg of magnetic Fe-MOF and His-nNOS/Fe-MOF were separately dispersed in 1 mL ethanol and then 5 µL samples were dropped onto carbon films of copper grids and dried overnight for observations.

**FT-IR analysis.**

The samples of His-nNOS, Fe_3_O_4_, magnetic Fe-MOF, His-nNOS/Fe-MOF and Flag-nNOS/Fe-MOF for Fourier transform infrared analysis (FT-IR) were prepared according to “Synthesis of magnetic Fe-MOF”, “Acquisition of nNOS and GFP-PSD95 proteins”, “Coordinative immobilization of nNOS on magnetic Fe-MOF surface” and “Preparation of PSD95-nNOS/Fe-MOF”. 3 mg samples were separately added in 300 mg potassium bromide and dried overnight at 50 °C in vacuum. The mixture was separately crushed and mixed well in an agate mortar, and then pressed into a pellet for measurements.

**Confocal scanning laser microscopy.**

Firstly, fluorescent dye Rhodamine B (RhB) was used to label His-nNOS. 3 µL RhB solution (1 mg/mL) and 300 µL His-nNOS PBS solution (1 mg/mL) were mixed uniformly and stirred at 4 °C for 12 h (120 rpm). After that, the uncombined RhB was removed by dialysis with a molecular weight cut-off of 8000-14000 Da for one day. The RhB-labelled His-nNOS was used for the preparation of PSD95-RhB-nNOS/Fe-MOF, which was the same as PSD95-nNOS/Fe-MOF, and the fluorescent images were observed by a confocal scanning laser microscopy. The red images were observed upon excitation at 594 nm while the green images were observed upon excitation at 488 nm.

**BET and XRD analysis.**

The samples of magnetic Fe-MOF and His-nNOS/Fe-MOF for Brunauer–Emmett–Teller (BET) specific surface area and X-ray diffraction (XRD) analysis were prepared according to “Synthesis of magnetic Fe-MOF” and “Coordinative immobilization of nNOS on magnetic Fe-MOF surface”. The samples were dried overnight at 50 °C in vacuum for determinations.

**VSM and TG analysis.**

The samples of Fe_3_O_4_, magnetic Fe-MOF, His-nNOS/Fe-MOF and PSD95-nNOS/Fe-MOF for vibrating sample magnetometer analysis (VSM) and thermal gravimetric analysis (TG) were prepared according to “Synthesis of magnetic Fe-MOF”, “Coordinative immobilization of nNOS on magnetic Fe-MOF surface” and “Preparation of PSD95-nNOS/Fe-MOF”. The samples were dried overnight at 50 °C in vacuum for determinations.

**Western blot analysis.**

The proteins eluted from PSD95-nNOS/Fe-MOF by 1×loading buffer were analyzed by western blotting analysis as well as free PSD95-nNOS. The proteins were separated by 10% acrylamide denaturing gels (SDS-PAGE) and transferred onto a polyvinylidene fluoride membrane. The load of the proteins eluted from PSD95-nNOS/Fe-MOF was three times more than that of free nNOS-PSD95. The primary antibodies were as follows: rabbit anti-PSD95 (1:1000, CST, PSD95 (D27E11) XP® Rabbit mAb #3450), rabbit anti-nNOS (1:1000, CST, nNOS (C7D7) Rabbit mAb #4231), mouse anti-Flag (1:2000, Proteintech, 66008-3-Ig). Internal control was used as follows: rabbit anit-β-actin (1:4000, Proteintech, 66009-1-Ig). Appropriate horseradish peroxidase-linked secondary antibodies were used for detection by enhanced chemiluminescence.


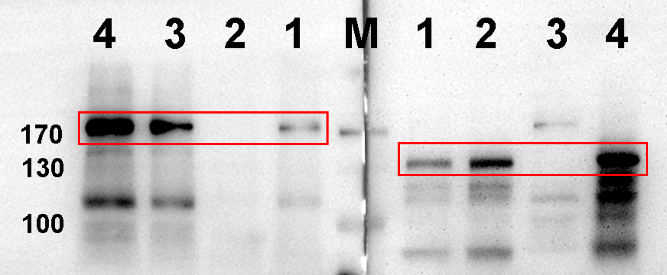


**Fig. S1** Western blotting analysis (M: marker, lane 1: PSD95-nNOS, lane 2: GFP-PSD95, lane 3: His-nNOS, lane 4: PSD95-nNOS/Fe-MOF, the lanes on the left of the marker were detected with nNOS-specific antibodies and the lanes on the right of the marker were detected with PSD95-specific antibodies)

**The pre-screening experiments on the compounds.**

We had performed the pre-screening experiments on the compounds to inspect their effect on the fluorescent intensity of PSD95-nNOS/Fe-MOF. We have investigated the quenching effect of the compounds on the fluorescence intensity of GFP-PSD95 in advance. The screened compounds (1 mM) were separately dissolved in a 96-well plate by 300 μL PBS buffer with 0.1% DMSO, and the following addition of 1.8 mg PSD95-nNOS/Fe-MOF was carried out. Additionally, 1.8 mg PSD95-nNOS/Fe-MOF were dispersed in 300 μL PBS with 0.1% DMSO as control. As shown in the Fig. S2, there were no significant changes in fluorescence intensities of the screened compounds compared with control group.


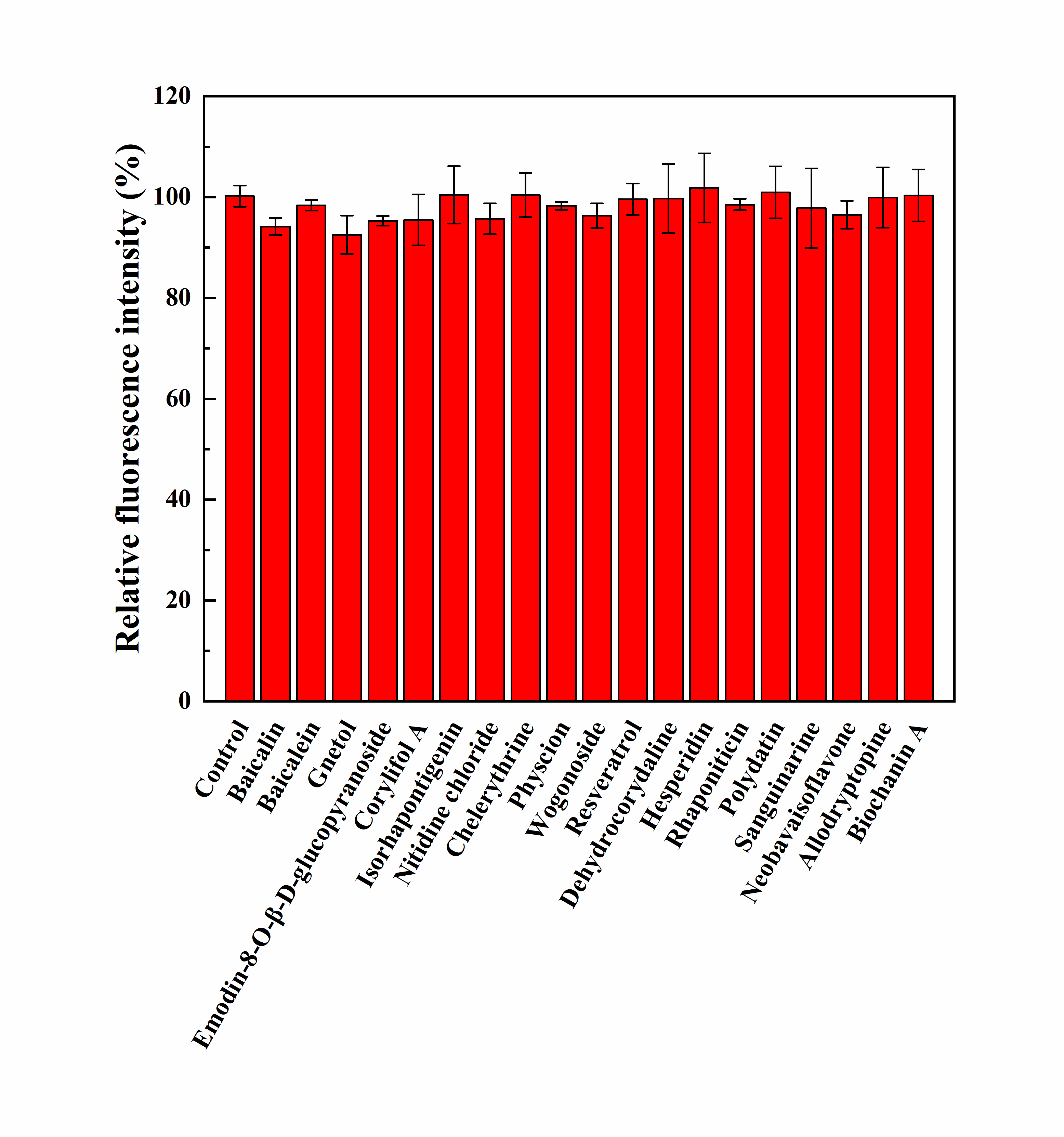


**Fig. S2** The quenching effect of nineteen compounds on the fluorescence intensity of GFP-PSD95

**Table S1. Comparison of the particle size of the materials to immobilize nNOS.**

| Materials | Particle size | Reference |
| --- | --- | --- |
| magnetic Fe-MOF | 10~20 nm | This work |
| Fe-COOH-MOF | 400 nm | [1,2] |
| UiO-66-NH_2_ | 100~200 nm | [3] |
| HKUST-1 | 530 nm (in H_2_O)  or 600 nm (in ethanol) | [4] |
| Zr-fum | 182 nm (in H_2_O)  or 132 nm (in ethanol) | [4] |
| Cu-BDC | 2000~3000 nm | [5] |
| SBA-15 | ＞200 nm | [6] |

**References**

1. Yin L, Wang Y, Tan R, Li H, Tu Y. Determination of beta-amyloid oligomer using electrochemiluminescent aptasensor with signal enhancement by AuNP/MOF nanocomposite. Microchimica Acta. 2021; 188: 53.
2. Huang YF, Liu QH, Li K, Li Y, Chang N. Magnetic iron (III)-based framework composites for the magnetic solid-phase extraction of fungicides from environmental water samples. Journal of Separation Science. 2018; 41: 1129-1137.
3. Mehta J, Dhaka S, Paul AK, Dayananda S, Deep A. Organophosphate hydrolase conjugated UiO-66-NH_2_ MOF based highly sensitive optical detection of methyl parathion. Environmental Research. 2019; 174: 46-53.
4. Roeder R, Preiss T, Hirschle P, Steinborn B, Zimpel A, Hoehn M, Raedler JO, Bein T, Wagner E, Wuttke S, Laechelt U. Multifunctional nanoparticles by coordinative self-assembly of His tagged units with metal organic frameworks. Journal of the American Chemical Society. 2017; 139: 2359-2368.
5. Yang L, Chen D, Wang X, Luo B, Wang C, Gao G, Li H, Li A, Chen L. Ratiometric electrochemical sensor for accurate detection of salicylic acid in leaves of living plants. RSC Advances. 2020; 10: 38841-38846.
6. Lu J, Wu JK, Jiang Y, Tan P, Zhang L, Lei Y, Liu XQ, Sun LB. Fabrication of microporous metal-organic frameworks in uninterrupted mesoporous tunnels: hierarchical structure for efficient trypsin immobilization and stabilization. Angewandte Chemie-International Edition. 2020; 59: 6428-6434.
